# Interlink between Inflammation and Oxidative Stress in Age-Related Macular Degeneration: Role of Complement Factor H

**DOI:** 10.3390/biomedicines9070763

**Published:** 2021-06-30

**Authors:** Sara Romero-Vazquez, Víctor Llorens, Alba Soler-Boronat, Marc Figueras-Roca, Alfredo Adan, Blanca Molins

**Affiliations:** Group of Ocular Inflammation: Clinical and Experimental Studies, Institut d’Investigacions Biomèdiques Agustí Pi i Sunyer (IDIBAPS), Hospital Clínic de Barcelona, 08025 Barcelona, Spain; sromerov@clinic.cat (S.R.-V.); vllorens@clinic.cat (V.L.); albasolerboronat@gmail.com (A.S.-B.); mafiguer@clinic.cat (M.F.-R.); amadan@clinic.cat (A.A.)

**Keywords:** age-related macular degeneration, inflammation, oxidative stress, complement factor H, retinal pigment epithelium

## Abstract

Age-related macular degeneration (AMD) heads the list of legal blindness among the elderly population in developed countries. Due to the complex nature of the retina and the variety of risk factors and mechanisms involved, the molecular pathways underlying AMD are not yet fully defined. Persistent low-grade inflammation and oxidative stress eventually lead to retinal pigment epithelium dysfunction and outer blood–retinal barrier (oBRB) breakdown. The identification of AMD susceptibility genes encoding complement factors, and the presence of inflammatory mediators in drusen, the hallmark deposits of AMD, supports the notion that immune-mediated processes are major drivers of AMD pathobiology. Complement factor H (FH), the main regulator of the alternative pathway of the complement system, may have a key contribution in the pathogenesis of AMD as it is able to regulate both inflammatory and oxidative stress responses in the oBRB. Indeed, genetic variants in the CFH gene account for the strongest genetic risk factors for AMD. In this review, we focus on the roles of inflammation and oxidative stress and their connection with FH and related proteins as regulators of both phenomena in the context of AMD.

## 1. Introduction to Age-Related Macular Degeneration (AMD) Pathophysiology

AMD is a multifactorial, chronic, and progressive degenerative disease of the central retina characterized by a decline in sharp central vision due to the atrophy of the macula [1]. It constitutes the leading cause of irreversible central visual loss in people older than 60 years old in high-income regions [2,3,4]. Meta-analyses of the numerous population-based studies of AMD have shown that the global pooled prevalence within an age of 45–85 years, of any form of the disease, is 8.69% [4]. Due to the increasing longevity and the extension of western diet and lifestyle, the projected number of cases is expected to increase and, by 2040, 288 million people suffering from any form of AMD are globally forecasted [4,5]. Age is the foremost risk factor for AMD development. Aging involves physiological changes at the inflammatory and oxidative stress levels that, in combination with genetic predisposition, improper diet and lifestyle, can trigger the onset and progression of AMD.

Although photoreceptor death is the ultimate factor responsible for vision loss, the germination of AMD occurs in the outer blood–retinal barrier (oBRB). The oBRB is composed of two cellular layers—the retinal pigment epithelium (RPE) and the choriocapillaris—and the Bruch’s membrane (BrM), a 2–4 µm acellular layer between them. This barrier tightly controls the transport of molecules, cells and nutrients from the systemic circulation to the outer retina in order to maintain tissue homeostasis and the immune privilege of the eye [6]. Whenever the oBRB becomes disrupted—where the odds increase with age—so does the local immune stage balance, and ocular diseases may arise. Early phases of AMD are usually characterized by the presence of drusen, which are deposits of extracellular matrix (ECM) beneath the basal lamina of the RPE, and RPE pigment irregularities. AMD can then evolve to the late forms of the disease, which include atrophic and neovascular (wet) AMD, both of which can lead to severe central visual impairment and legal blindness. Approximately 85–90% of AMD patients suffer from the atrophic or non-exudative condition [7]. In this case, RPE atrophy may result in photoreceptors and choriocapillaris loss. Instead, neovascular (exudative) AMD, present in 10–15% of patients, is characterized by exacerbated vascular growth and invasion of neovessels through the BrM into the retina.

**Figure 1:**
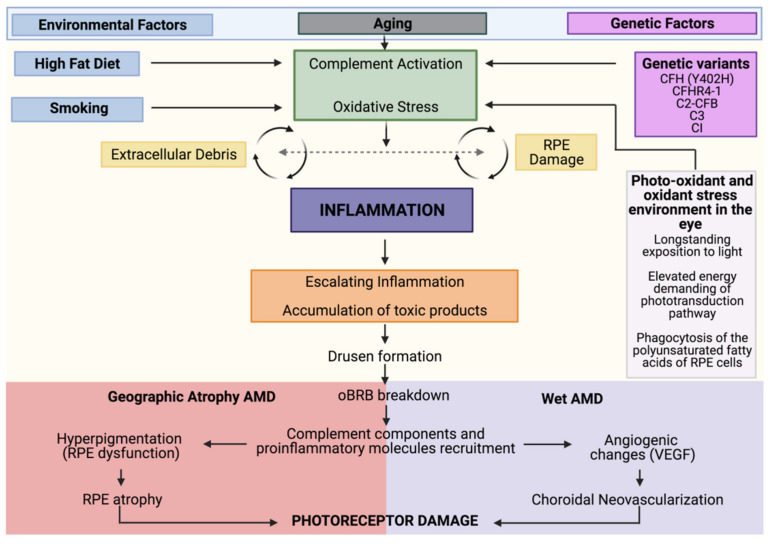
AMD pathobiology. Integrated model of AMD pathogenesis with a central involvement of inflammation. As individuals age, they experience increased oxidative stress, and complement dysregulation. The convergence of genetic (in magenta) and environmental (in blue) factors leads to a state in which the accumulation of toxic elements leads to a sustained activation of these pro-inflammatory and damaging pathways that culminates in advanced AMD. In the case of geographic atrophy (GA), sustained damage to the retinal pigment epithelium (RPE) leads to RPE and choriocapillaris degeneration. In neovascular AMD, breakdown of the outer blood–retinal barrier (oBRB) results in immune cell trafficking into the retina, which drives vascular endothelial growth factor (VEGF)-dependent neovascularization. Both forms ultimately result in photoreceptor damage and visual impairment.

Despite being the primary cause of vision loss in developed countries, the mechanisms by which the different risk factors and pathways converge towards AMD are uncertain and therefore drug discovery is challenging. Anti-angiogenic therapies that target vascular endothelial growth factor (VEGF) have been successful in substantially improving central vision in approximately 30% of patients. However, most treated patients still suffer from visual impairment as they develop fibrosis and atrophy, and more than one-third of them show long-term loss of effect. Most concerning is that there are no approved therapies for geographic atrophy (GA), the advanced stage of non-exudative AMD, mainly due to the lack of suitable molecular targets. Importantly, these advanced stages of AMD are not mutually exclusive. In addition, long-term treatment of neovascular AMD with VEGF-targeted therapy is associated with the development of GA. Genetic polymorphisms that are associated with AMD confer a similar statistical risk of developing both forms of AMD, which indicates that there are many shared underlying pathological mechanisms. Although still not completely understood, the interlink between oxidative stress, RPE dysfunction, genetic variants, cellular senescence, and altered immune response shapes the pathobiology of AMD (Figure 1) [8].

### Genetic and Environmental Risk Factors

Although age is the primary risk factor, there are other genetic and environmental factors that influence AMD susceptibility. Genetic, epidemiological, and histopathological studies have linked the immune system, specifically the complement cascade, to AMD [9]. Likewise, oxidative stress-induced RPE damage has been reported to be an important driving factor [1]. Environmental and lifestyle risk factors related to enhanced oxidative stress and proinflammatory state contribute to AMD development. Smoking increases the risk of neovascular AMD both in females and men [10]. In two cross-sectional studies, the chronic exposure to cigarettes (at least 1 cigarette per day for a year) was correlated with the presence and severity of AMD. Smoke increased the odds ratio (OR) of GA by around 2.5% and neovascular AMD by 3.5%. Interestingly, individuals with 20-smoke free years presented OR comparable to non-smokers [11,12]. In addition, in vitro studies have also reported alterations in RPE function and in complement and oxidative stress activation after cigarette smoke exposure [13,14,15]. Raised levels of cholesterol, elevated body-mass index and gut dysbiosis have also been associated with AMD susceptibility [1,16,17]. Dietary interventions with carotenoids, oral supplementation with high levels of antioxidants and minerals, or high intake of omega-3 fatty acids and fish seem to slow the course of the disease [18,19]. Light and photosensitization reactions may also play a role in the development of AMD via the synthesis of reactive oxygen species (ROS), with consequent damage to the RPE and BrM [20]. Finally, chronic systemic disorders such as atherosclerosis [21], diabetes [22], and cardiovascular diseases [23] contribute to the risk for AMD development.

Twin studies about the genetic burden of advanced AMD have shown that up to 70% may correspond to inheritance component, and only 30% seems to rely on environmental causes [24]. A variety of complement pathway-associated gene variants, such as *complement factor H* (*CFH*), *factor B*, the complement components *C2*, *C3*, and *C5*, and the *ARMS2/HTRA1* genes have associations with AMD pathogenesis [25]. Genes related to neovascularization, such as *VEGF*, *TIMP-3*, and *Fibulin 5*, and lipoproteins (*ApoE*, *hepatic lipase*, *CETP*, and *CD36*) have also shown associations to AMD [1]. In 2016, Fritsche and colleagues presented the largest genome-wide association study to date, in which they reported 45 common single-nucleotide polymorphisms and 7 rare variants independently associated with AMD risk [26]. Noteworthy, as will be discussed in the following sections, a common polymorphism in the *CFH* gene, a key regulator of the complement system, is one of the most reported contributors to AMD susceptibility. This variant is present in 35% of the Caucasian European population and several genetic studies have significantly associated this polymorphism with a major susceptibility to AMD [27,28,29,30]. It increases the risk ~2–4-fold for heterozygous and up to 7-fold for homozygous individuals. A plausible hypothesis is that the *CFH* risk polymorphism displays an altered capacity to restrain both complement cascade and oxidative stress damage.

## 2. Oxidative Stress and AMD

Oxidative stress increases with age due to two complementary phenomena. The increase in intracellular oxidizing products that occurs with age is accompanied by a reduced anti-oxidant capacity and cellular waste removal function. Oxidized proteins become partially unfolded and tend to form aggregates inducing cell activation and apoptosis. Furthermore, the presence of oxidized phospholipids in cell membranes constitutes an oxidation-specific epitope, danger-associated molecular patterns (DAMPs), which are self-marker signals of cellular damage for immune cells. Recruitment and activation of macrophages and release of natural antibodies against oxidized lipoproteins are the downstream consequences that facilitate the turnover of unhealthy cells and maintain homeostasis [31]. 

The retina is particularly prone to suffer from photooxidation and oxidation due to its prolonged exposure to light and its high metabolic activity. When oxygen absorbs enough light energy it converts into singlet oxygen, which remains reactive for a relatively long period. In the retina, singlet oxygen can lead to the peroxidation of polyunsaturated fatty acids (PUFA) of the membrane of photoreceptors’ outer segments (POS), transforming them into organic radicals. Therefore, long light exposure increases the formation of free radicals that, eventually, leads to the oxidized lipid products that populate the aged RPE monolayer [32]. Recent studies have demonstrated that photooxidative light modulates the inflammatory response in RPE cells [33]. Exposition to blue light has been demonstrated to increase cell apoptosis and to impair autophagy in RPE cells which may be mediated by an increase in ROS [34]. Oxidized proteins and lipids also produce dysfunction in the lysosomal system. The RPE is largely post-mitotic and copes with a huge proteolytic burden. Thus, this removal of cellular debris is instrumental for normal cellular function [35]. A dysfunctional lysosomal system provokes further accumulation of oxidized molecules and it is associated with AMD [14,36,37]. Proteomic analysis of the drusen and outer retina found that oxidized lipoproteins are more abundant in AMD patients than in healthy donors. Interestingly, also systemic oxidized lipoproteins are increased in AMD patients [38]. 

On the other hand, the retina is considered the most metabolically active tissue of the body due to the elevated energy demand for the phototransduction pathway [39]. Choriocapillaris, which supplies constant nutrients and oxygen to the outer retina, accounts for 85% of the blood supply of the retina [40]. In fact, both RPE cells and photoreceptors’ inner segments are enriched in mitochondria, whose respiratory process is an important producer of free radicals [39,41]. 

Increased oxidative stress in the retina also emerges from the phagocytosis of the PUFA of the POS disk membranes by the RPE. This recycling process is critical in the phototransduction pathway and results in aged POS disk membranes continuously renewed (roughly, 10% of POS are removed each day [42,43]). This process also results in the accumulation of metabolic debris from incomplete POS digestion that generates lipofuscin, an end-product highly relevant in the oxidative stress-mediated damage in AMD. Lipofuscin comprises a group of autofluorescent lipid–protein aggregates (e.g., bis-retinoid pyrimidine (A2E), carboxyethylpyrrole protein, malondialdehyde (MDA), 4-hydroxynonenal, etc.). Given that POS digestion is continuously required throughout life, lipofuscin accumulation increases with age, with ~1% of the cytoplasmic of RPE volume covered by lipofuscin in our first decade of life, compared to ~19% in our 80 s [15]. Lipofuscin reaches its highest concentration in the macula and, therefore increases the susceptibility to light-induced damage in the RPE [32,44]. Oxidized products of lipofuscin such as MDA, A2E and carboxyethylpyrrole protein are present in the RPE, but also in soft drusen and produce ROS when exposed to light [45]. These age-related fluorophores are strong immunogenic factors that trigger immune responses that provoke the release of antibodies and autoantibodies [46]. Specifically, A2E induces DNA damage and apoptosis through oxidative stress mechanisms in RPE cells when exposed to blue light radiation [47,48,49]. Similarly, MDA, generated as an end-product of PUFA oxidation, is a widely and reliable marker of light-induced oxidative stress that has been associated with AMD [50]. Indeed, MDA can cause RPE damage and MDA-modified proteins are known to induce inflammatory responses and are recognized by innate immunity [51,52].

## 3. Inflammaging and Cellular Senescence in AMD

In order to preserve a functional neuroretina, it is necessary to maintain an immune tissue tone (named “para-inflammation”) able to protect the eye against over-inflammation and to keep control over cellular stress. This homeostasis is accomplished with immunosurveillance of immune-resident cells (microglia and resident macrophages), and non-immune cells (Muller glia and RPE cells), and a baseline deposition of complement proteins and antibodies [53]. However, with age, a switch in this basal grade inflammation may occur, becoming over-active and resulting in a chronic inflammatory stage. This phenomenon, known as “inflammaging”, characterized by a senescent phenotype of immune cells, is present in age-related conditions [53,54]. 

Analysis of the basal inflammatory stage of aged humans (older than 65 years old) showed both a decrease in autophagy and an increase in pro-inflammatory cytokines and acute phase reactants compared to younger individuals [54]. Likewise, phagocytosis of apoptotic cells by monocytes and dendritic cells is diminished with age [54]. Although the mechanisms underlying inflammaging have not been fully elucidated; changes in the expression of pattern recognition receptors and activation of pattern recognition receptors by DAMPS seem to be involved. The downstream release of pro-inflammatory cytokines is the consequence of this mistaken signaling pathway [54]. In fact, damaged DNA released from necrotic or apoptotic cells, together with oxidation-specific epitopes also expressed on apoptotic cell surfaces formerly discussed, are also DAMPS, able to trigger the immune response. 

Cellular senescence is a natural phenomenon that comes along with aging and has beneficial effects such as limiting tissue damage and tumor-suppressor properties. However, persistent senescence creates a chronic inflammatory environment that can trigger age-related diseases. The irreversible cell-cycle arrested cells display an altered genetic profile, increased cell size, altered morphology and a senescence-associated secretory phenotype. This phenotype, frequently present in chronic diseases, involves proinflammatory cytokines, matrix-remodeling proteases and growth factors [37]. Another important hallmark of senescence is cellular protein aggregate-accumulation (e.g., lipofuscin), which coincides to be highly relevant in the oxidative-stress mediated damage in AMD. Therefore, in addition to telomere erosion, oxidants and molecules mediating oxidative stress damage can trigger cellular senescence, inducing stress-induced premature cellular senescence [37,55]. Indeed, ocular inflammaging is a key feature of AMD [56].

## 4. The Complement System in AMD

The identification of AMD susceptibility genes encoding complement factors and the presence of complement and other inflammatory mediators in drusen support the concept that local inflammation and immune-mediated processes play a key role in AMD pathogenesis. The complement system is a protein cascade composed of more than fifty proteins, found in both the fluid phase and bound to cell membranes. The main role of the complement system is to recognize and mediate the removal of pathogens, debris and dead cells [57]. Proteins of the complement system can be rapidly converted into active forms via a proteolytic cascade triggered by any of the three activating pathways: the classical pathway, the lectin pathway, and the alternative pathway (AP) [58]. All three pathways converge in the formation of the C3 convertase, which cleaves C3 into the anaphylatoxin C3a, ant C3b. C3b is then needed to form the downstream C5 convertase, the complex responsible for the cleavage of C5 into the second anaphylatoxin C5a and C5b. C5b is then required for the terminal pathway that ends with the formation of the membrane attack complex (MAC), which induces cell lysis in the target material [58].

The first thorough screening for drusen components provided additional support for the concept that immune processes may be involved in drusen biogenesis. Drusen are immunologically active deposits containing oxidative lipids, lipofuscin, acute-phase reactants, immunoglobulins and proteins involved in the complement and immune response [38,59,60], that may contribute to oBRB dysfunction. On the other hand, unlike other capillary beds, the choriocapillaris is particularly prone to the deposition of MAC, the final step of the complement cascade, and there is increased accumulation of MAC in BrM and choriocapillaris in elderly individuals and in AMD patients [61]. 

As discussed above, a striking feature of AMD is the presence of chronic inflammation in the eye. Complement activation leads to the recruitment and activation of immune cells mediated by complement activation in local tissues and the release of the anaphylatoxins C3a and C5a. The immune cells involved in AMD comprise not only retinal resident microglia cells, but also circulating lymphocytes and monocytes/macrophages and mast cells [62,63]. Indeed, the stimulation of monocytes by C3a can lead to interleukin (IL)-1β secretion and NLRP3 inflammasome activation [64] and both C3a and C5a cause an increase in NF-kB signaling in monocyte-derived dendritic cells [65]. Besides classical immune cells, complement activation can also stimulate RPE cells into secreting a range of inflammatory cytokines, such as IL-6, IL-8 and monocyte chemotactic protein-1, further contributing to oBRB dysfunction [66]. Moreover, microglia also modulates the activity of RPE cells, providing even more cellular feedback in the outer retina [67].

## 5. C-Reactive Protein

C-reactive protein (CRP) is the prototypical acute-phase reactant that belongs to the pentraxin family. In clinical practice, CRP has been classically considered an unspecific inflammation biomarker since its serum levels quickly raise up from 1–5 mg/L in the physiological state to 1000-fold within hours in response to inflammation, infection or tissue injury mediated by inflammatory cytokines (mostly IL-6 and IL-17). It is primarily secreted by the liver, although some extrahepatic synthesis has also been reported [68]. Among the multiple functions ascribed to CRP are the activation of the classical complement pathway and inactivation of the AP [69]. CRP is considered to be a serum biomarker for chronic inflammation, heart disease and, more recently, also AMD [21,70]. In a prospective longitudinal study, elevated levels of CRP in serum (>10 mg/L) were positively and significantly associated with the progression of AMD [70].

In plasma, CRP typically exists as a cyclic, disk-shaped pentamer (pentameric CRP, pCRP) composed of five noncovalently linked subunits of 23 kDa [71]. However, pCRP can undergo dissociation into its subunits, acquiring distinct biological functions. Oxidative stress and bioactive lipids from activated or damaged cells can dissociate pCRP into its 23-kDa subunits [72,73,74] through a mechanism that is dependent on lysophosphatidylcholine exposure after phospholipase A2 activation [74]. This alternative conformation of CRP, termed monomeric CRP (mCRP), has different antigenicity-expressing neoepitopes and represents the tissue-based insoluble form of CRP. Unlike pCRP, mCRP displays a proinflammatory phenotype in several cell types [73,75,76].

In a local scenario, CRP has been detected in macular drusen and in subepithelial deposits [59,60]. Chirco and colleagues showed a link between AMD risk and elevated levels of mCRP, but not pCRP, in the choroid. In their work, Chirco et al. also showed that mCRP exerts an inflammatory effect on choroidal endothelial cells, as it increased cell migration and upregulated inflammatory gene expression including *ICAM1*, suggesting a role for mCRP in promoting inflammation in the choroid. They hypothesized that the accumulation of mCRP in the choroid should be in the early events of AMD development, as it is present prior to the onset of the disease [59]. In line with this study, we showed that mCRP, but not pCRP, induces oBRB disruption, at least in vitro. Monomeric CRP disrupts the RPE barrier function by disturbing the expression and distribution of tight junctions and increasing paracellular permeability [77]. Likewise, mCRP upregulates the production of IL-8 and monocyte chemotactic protein-1 in RPE cells. Moreover, mononuclear cell migration increased when exposed to conditioned medium from mCRP-treated RPE cells, but not in pCRP- or control-treated conditioned media [78]. Interestingly, topological localization of mCRP determines the RPE pro-inflammatory response as RPE disruption is only observed when mCRP is exposed to the apical domain [77]. These findings together with the fact that there is no transcription of *CRP* in the retinal tissue, led us to study how CRP isoforms could accumulate in the subretinal space. Using a Transwell model we found that both pCRP and mCRP can cross endothelial cells and reach the RPE in vitro. Interestingly, mCRP, but not pCRP, is able to cross a compromised RPE monolayer but not a highly polarized RPE monolayer. Alternatively, mCRP can also originate from the dissociation of pCRP in the surface of lipopolysaccharide-damaged RPE [77]. Whether these in vitro findings translate to disease or not require validation but there is increasing evidence that supports mCRP contribution to AMD progression.

## 6. Complement Factor H and Related Proteins

**Figure 2:**
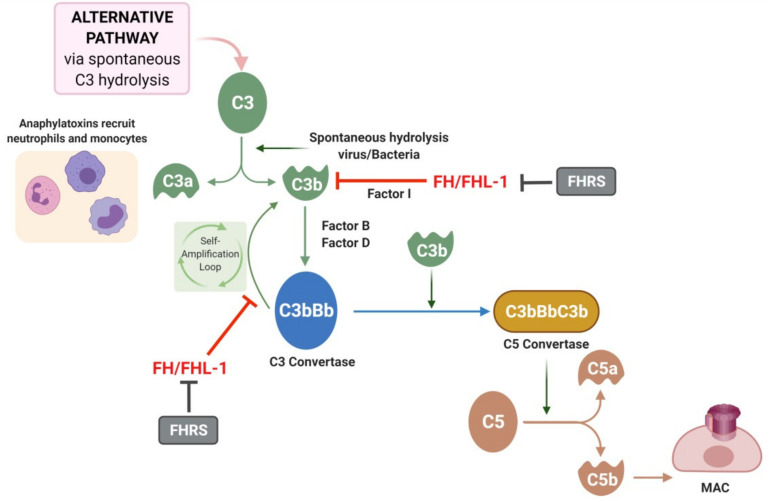
Alternative pathway and role of complement Factor H (FH). The alternative pathway is triggered by the spontaneous hydrolysis of C3 which forms C3a and C3b. C3b is able to interact with factor B and factor D creating the C3bBb molecule also known as C3 convertase. The C3 convertase of the alternative pathway triggers an amplification loop, with further hydrolysis of C3. Ultimately, C3b can join the C3 convertase to form C3bBbC3B or C5 convertase, which cleaves C5 to C5a and C5b. C5b leads to formation of the membrane attack complex (MAC). FH/FHL-1 can interact with C3b molecules or with the C3 convertase complex avoiding the amplification loop. FH-related proteins (FHRS) compete with FH for C3b binding and inhibit Factor I-mediated C3b cleavage.

Complement Factor H (FH) is a ~155 KDa plasma glycoprotein and the main regulator of the AP of the complement that confers protection to the ECM [79]. It is mainly secreted by the liver and constitutes the most abundant complement protein in plasma (~300 µg/mL) although the levels slightly decrease with age [80]. Besides hepatocytes, endothelial and RPE cells also synthesize FH [79]. FH binds to molecules naturally present in host tissues inhibiting the activation and amplification of the complement cascade through the AP [81]. The AP of the complement system presents a unique characteristic, since unlike the other initiating pathways it is constitutively active at low levels due to spontaneous hydrolysis of C3 (Figure 2). The AP starts, after C3 spontaneous cleavage, with tissue deposition (in cells or in ECM) of C3b. If C3b interacts with factor B, it forms a complex that can be cleaved by factor D producing C3bBb convertase that promotes forward the amplification loop by cleaving more C3. The downstream consequences are the release of anaphylatoxins C3a and C5a and the deposition of MAC. Alternatively, C3b can interact with FH allowing cleavage by factor I (FI) and preventing the complement response. FH is also able to dissociate the C3 convertase (C3bBb) avoiding the amplification loop. The feasibility to discriminate host surfaces from non-self surfaces constitutes a cornerstone in the immune system. In the case of FH, this ability lies in the binding to glycosaminoglycans (GAGs), long unbranched chains of proteoglycans [79]. GAGs are abundant on host tissues, constituting a self-marker to avoid improper amplification of the AP. Meanwhile, pathogens do not usually express GAGs and thus, AP cannot be inhibited [81]. Heparan sulfate (HS) is a ubiquitously GAG and a major FH ligand in the retina [82]. 

**Figure 3:**
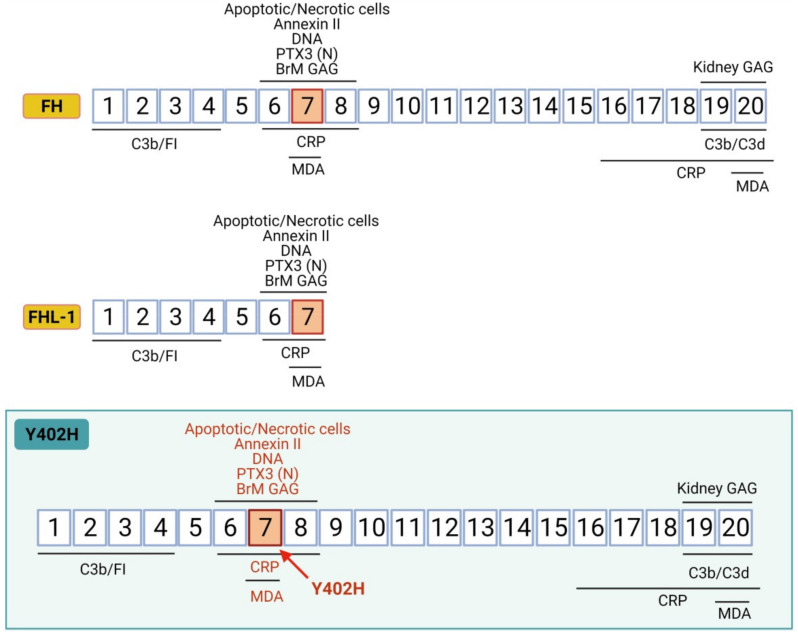
Complement factor H structure. The CFH gene encodes a single chain of 20 complement control protein domains (CCPs). CCPs 1–4 and CCPs 19–20 are C3b binding regions; CCPs 6–8 are glycosaminoglycan (GAG)-binding regions in BrM; CCPs 19–20 are GAG-binding regions in kidney; CCPs 6–8 and CCPs 16–20 are C-reactive protein (CRP) binding regions and CCPs 7 and CCP 20 are malonaldehyde (MDA) binding regions. Y402H variant implies a tyrosine to histidine substitution at amino acid 402 in CCP7, the domain involved in binding to GAGs-BrM, CRP, MDA, oxLDL, and apoptotic cells. FHL-1 is a naturally occurring truncated form of FH that predominates in BrM and encodes the first seven FH CCPs. Therefore, FHL-1 maintains regulatory function and binding to CRP, MDA and BrM-GAG, and it is also affected by the Y402H polymorphism that lies on CCP7 domain.

*CFH* gene is located on chromosome 1q32 in the “regulators of complement activation” gene cluster [83]. *CFH* encodes a single chain of 20 complement control protein (CCP) domains that display different functions due to both diversity in sequence and to interactions between neighboring CCPs which produce diverse spatial orientation arrangements [81] (Figure 3). Each CCP comprises ~60 residues and folds into a β-strand-rich domain. CCPs are also referred to as short consensus repeat or sushi domains, and are flexibly linked by three to eight amino acids residues. The first four N-terminal CCPs of FH confer regulatory activity by mediating the binding to C3b and FI [84]. C3b, via its C3d region, also binds to the C-terminal CCPs 19–20 of FH. Based on nuclear magnetic resonance spectroscopy and X-ray studies, CCPs 6–8 and CCPs 19 and 20 are known to be the two major GAGs-binding regions. However, they exhibit different tissue-specificity, being CCP7 the functional unit responsible for surface anchoring to BrM, while CCP20 accomplishes its function in the kidney [79,81,85]. The CCPs 6–8 and/or 19–20 regions also support the interactions with a range of ligands including pentraxins (CRP and PTX3), the lipid peroxidation product MDA, apoptotic/necrotic cells (via annexin II and DNA), ECM proteins, and Apolipoprotein E [81]. 

FH-like 1 protein (FHL-1) is a ~49 KDa naturally occurring truncated form of FH, arising from the *CFH* gene alternative splicing. FHL-1 comprises the first seven CCPs, and thus retains the complement regulatory function and binding sites for BrM-GAGs, pentraxins, and MDA. Both FH and FHL-1 have been detected in vitreous humor and local transcription by RPE cells has been also reported [86]. However, due to their differences in size (155 vs. 49 KDa), they show dissimilar diffusion properties and distribution across the oBRB. Clark and collaborators showed that the BrM divides complement regulation between the choroid and the retina [79,82,86]. They studied the permeability of BrM to different complement proteins. Large molecular weight, glycosylation and a great net positive charge are obstacles to the mobility across the BrM. While FH surrounds drusen deposits, FHL-1 is able to penetrate inside them. Moreover, unlike FH, FHL-1 can passively diffuse through the BrM. Thus, FH is the predominant form in the choroid, while FHL-1 is the predominant regulator in the BrM-RPE [82]. The immobilization of FH and FHL-1 is mediated by HS GAGs. However, HS in the BrM decreases with age, with a reduction of 50% in old versus young donors (average 82 vs. 32 years) [87], impacting local complement activation management [87]. 

Downstream of the *CFH* gene are five complement factor H-related genes (*CFHR1-5*) and in contrast to the FH and FHL-1 regulatory proteins, the five FH-related proteins (FHR1-5) are believed to act as positive activators of the complement system [83,88]. The FHR proteins compete for binding to C3b, but they do not share the FI co-factor domain possessed by FH and FHL-1, and therefore actively prevent FI-mediated C3b breakdown, leading to increased complement turnover [88]. A GWAS study recently identified an intronic variant in the *CFHR-4* gene that was associated with increased systemic complement activation and increased AMD risk [89]. Afterwards, Cipriani and coworkers demonstrated that increased circulating levels of FHR4 are strongly associated with AMD and provided evidence that FHR-4, which accumulates in choriocapillaris, competes with FHL-1 to bind C3b molecule. Therefore, although FH/FHL-1 are the main complement regulators in the choriocapillaris/BrM, excessive FHR-4 may compete with FH/FHL-1 in C3b binding leading to unrestrained inflammation and predisposing to AMD [90].

In the last few years, several research studies have identified various non-canonical functions of FH, highlighting a mutual relationship between the complement system and oxidative stress. Not only are complement levels changed in response to stress, but also a functional complement system is needed for a proper cellular response to stress. FH, either from local or systemic sources, plays a protective role against oxidative stress in RPE cells and this non-canonical function is altered by AMD-associated polymorphisms of the *CFH* gene [91,92]. Indeed, exogenously applied recombinant FH protein protects RPE cells from damage induced by the exposure of lipid oxidation products, reducing the levels of cell death, complement activation, membrane and mitochondrial damage [91]. Moreover, loss of endogenous FH in RPE cells renders RPE cells more vulnerable to oxidative stress, leading to increased lipid peroxidation and reduced viability [92]. On the other hand, FH has been found to be present in human plasma in two different redox forms, with the reduced form higher in early AMD and the oxidized form being higher in late AMD [93]. Interestingly, the two redox forms have different functions: the oxidized form mediates more efficiently FI-mediated cleavage of C3, while the reduced form protects RPE cells from oxidative damage [93].

FH also binds to native and oxidized LDL (oxLDL) [31] and this binding is important in the modulation of the inflammatory response. Indeed, oxLDL upregulates IL-8, tumor necrosis factor-α and VEGF in RPE cells and macrophages, and the binding of FH to oxLD mitigates such induced inflammation [31]. Moreover, FH also reduces the uptake of oxLDL by RPE cells [94]. Notably, these functions are abolished in the presence of the 402H variant or in the presence of FHR proteins, which are antagonists of FH [31,94].

### 6.1. Y402H Polymorphism: Unrestrained Inflammation and Oxidative Stress

The AMD-associated *CFH* variant Y402H encompasses a tyrosine (Y) to histidine (H) substitution at amino acid 402. The polymorphism lies on CCP7, the domain involved in FH binding to HS in the ECM of the BrM, and also to CRP, MDA, oxLDL, and apoptotic cells (Figure 3) [81]. It has been proposed that the Y402H variant impacts the binding affinity of FH to such ligands, potentially leaving local pro-inflammation and oxidative stress unconstrained [95,96,97].

#### 6.1.1. Impaired Binding to CRP

FH is known to bind CRP. Johnson et al. showed that choroidal immuno-staining of CRP was significantly higher in eyes carrying the Y402H variant of *CFH* [98]. Because the high-risk allele affects the binding of FH to CRP [99], deficient FH binding could potentially increase the pro-inflammatory activity of CRP in choroidal tissue, contributing to AMD pathogenesis. Additionally, Bhutto and colleagues found an inverse relationship between CRP and FH levels in macular tissue from patients with advanced AMD as compared to age-matched control individuals [100]. In AMD patients, BrM, drusen, and choroidal vessel walls, all showed increased labeling of CRP and decreased labeling of FH compared to controls.

Two separate binding sites for pCRP are located on domains SCR4-6 and SCR16-20, respectively [101]. However, FH shows strong binding to mCRP, rather than to the native form [102,103]. Moreover, we showed that the “non-risk” FH variant can effectively bind mCRP to dampen mCRP proinflammatory activity. Notably, FH from AMD patients carrying the risk polymorphism for AMD shows an impaired binding to mCRP and, therefore, its proinflammatory effects remain unrestrained [78]. In line with our findings, Chirco et al. also showed that mCRP levels are elevated in the choroid-RPE of individuals with the high-risk *CFH* genotype [104], which could thus sustain chronic inflammation contributing to the progression of AMD in individuals with the risk Y402H FH variant.

#### 6.1.2. Impaired Binding to GAGs

Y402H polymorphism alters the sulfation pattern recognized in HS [105,106,107]. The non-risk form of FH recognizes a broader range of HS structures, whereas the risk form requires more highly sulfated HS for binding, reducing its ability to interact with HS within the human BrM [106,108]. The restricted binding specificity of the disease variant of FH may lead to its impaired association with the BrM, and this would allow inappropriate complement activation driving disease progression [107,108]. Moreover, the substantial reduction in the amount of HS in BrM with age could likely explain the larger difference in the relative binding activities of the 402H and 402Y variants in old versus young tissues [87].

#### 6.1.3. Reduced Protection from Oxidative Damage

Two active mediators of oxidative stress and lipid peroxidation, MDA and oxLDL, respectively, bind FH through CCP7. Thus, the Y402H polymorphism may impact as well their binding affinity to FH. In 2011 Weismann et al. identified FH as a major MDA-binding protein that can block both the uptake of MDA-modified proteins by macrophages and MDA-induced proinflammatory effects. Indeed, the Y402H risk variant markedly reduced the ability of FH to bind MDA [109]. In line with these results, Aredo and coworkers showed that a chimeric transgenic mouse strain carrying the CFH Y402H presented increased oxidative stress, via MDA in response to ageing, light exposure and hydroquinone diet [110]. Similarly, FH from homozygous Y402H risk variant shows lower affinity to bind oxLDL, leading to lower protection against activation of oxidative-mediated events in RPE cells [31,94].

## 7. Contribution of Systemic vs. Local FH and Related Proteins to AMD Pathogenesis

The retina expresses several complement proteins, which may render it more susceptible to complement-mediated damage. Indeed, complement dysregulation and altered complement production occur locally with advancing age and in AMD [111]. Concurrently, systemic complement activation is also strongly associated with AMD and retinal deposition of circulating complement components or defective complement regulatory proteins may contribute to disease [112]. Both locally and systemically produced complement may therefore play a role and compound one another in AMD. In particular, FH and FHL-1 are expressed in the liver but also in the RPE. The fact that the Y402H polymorphism does not impact circulating FH may support the greater contribution of local versus systemic complement activation. Indeed, Khandhadia et al. showed in liver transplant patients that while circulating CFH protein allotype is completely determined by donor liver *CFH* genotype, AMD risk in liver transplant patients is associated with recipient rather than donor *CFH* genotype [113], suggesting that locally produced FH plays a greater role than circulating FH in AMD pathogenesis. While the local contribution of FH/FHL-1 may be more important than its systemic contribution, this may not be the case for FHR-4. The recent work of Cipriani et al. showed that systemic FHR-4 levels are increased in AMD [90]. While no *CFHR-4* transcription is found in the eye, CFHR-4 accumulates in the choriocapillaris, BrM and drusen, and can compete with FH/FHL-1 for C3b binding, preventing FI-mediated C3b cleavage, highlighting the role of its systemic contribution to AMD pathogenesis. A better understanding of the relative contribution of local and systemic FH and related proteins would clarify which source is more important and would also help how to target complement activation in AMD.

## 8. Targeting Complement Factor H and Related Proteins for Treating AMD

Due to the variety of mechanisms that converge into the pathobiology of AMD, several pathways are being targeted in phase I/II/III clinical trials, including anti-inflammatory agents, complement system inhibitors, visual cycle modulators, antioxidants, reducers of toxic by-products, stem cell therapies, and gene therapies [114]. The strong genetic and biological evidence that overactivation of the complement system, in particular, the AP, is a major driver of AMD pathogenesis, has increased the interest in complement-mediated therapeutic strategies. Specifically, given the key role of FH/FHL-1 and related proteins in the regulation of complement activation, but also in protecting from oxidative stress and inflammation, makes them promising candidate targets to treat AMD.

Genetic variants in chromosome 1q31.3 in the “regulators of complement activation” gene cluster, encompassing the CFH and CFHR1-5 genes are major determinants of AMD susceptibility. Rare variants in CFH, generally result in a familial, early-onset condition, affecting the function of both FH and FHL-1 [80,81]. Notably, the Y402H “risk” variant results in a reduced binding of FH/FHL-1 to several ligands (HS, CRP, MDA, oxLDL, etc.) that results in local uncontrolled inflammatory and oxidative stress response [95,96]. Genetically susceptible patients could be treated with supplementation strategies to overcome reduced FH/FHL-1 function. In this regard, Gemini Therapeutics has successfully concluded a phase I clinical trial investigating the monthly intravitreal injection of a full-length recombinant FH (GEM103) in patients with GA [96]. However, FH cannot cross the BrM [82], so it remains to be seen whether enough FH is able to reach the underlying ECM of the choriocapillaris. Alternatively, gene therapy targeting RPE cells to express the non-risk FHL-1 variant in AMD patients carrying the Y402H polymorphism could overcome the bioavailability issue. RPE cells could basolaterally release the therapeutic targeting only the local complement system. Targeting FHR-4 may also represent a future therapeutic avenue to explore, as high circulating levels of FHR-4 are associated with AMD risk and a variant in the CFHR4 gene has recently been associated with increased complement activation and AMD risk. Lowering systemic FHR-4 levels could be achieved using antibodies or other agents that block/sequester the protein or by anti-sense targeting of hepatic FHR-4 synthesis.

The efficacy of these strategies would be enhanced by stratification of patients based on their genetic susceptibility (variants in chromosome 1q31.3) and their acquired risk factors in order to target those who will be most sensitive to the respective treatments. Indeed, this has been postulated as one of the reasons for the failure of previous clinical trials, such as the anti-FD antibody Lampalizumab [115].

Given the multifactorial nature of AMD one should also consider that at the time of therapeutic intervention, RPE cells have already undergone degeneration. Therefore, it is reasonable to consider a combinational therapy targeting the FH pathway and RPE cells at the same time. For instance, several antioxidants agents and potential mitochondrial protective substances have been suggested as cytoprotective options for RPE cells in AMD [116,117].

## 9. Concluding Remarks

In this review, we discussed the structure/function relationships of complement FH and related proteins as regulators of inflammation and oxidative stress in the pathophysiology of AMD. The reduced ability to control the balance between pro- and anti-inflammatory signals associated with aging might promote a switch to chronic inflammation in the macular tissue that would be also accompanied by an increased oxidative stress state. FH, as the main regulator of the AP of the complement system, is able to regulate both inflammatory and complement-related responses but is also able to protect from oxidative stress through non-canonical pathways. However, in genetically susceptible patients, with alterations in the CFH and CFHR1-5 genes, FH may not be able to control exacerbated chronic inflammation and tissue damage. FH/FHL-1 from patients carrying the Y402H risk variant may be unable to localize HS to BrM and to dampen mCRP- and MDA/oxLDL-mediated proinflammatory and oxidant-related activities, respectively. Likewise, in patients with increased FHR4, excessive FHR4 may compete with FH/FHL-1 leading to overactivation of the complement system and reduced ability to protect from oxidative stress in the macular tissue. These scenarios combined with aging-associated processes, such as HS loss and an increased proinflammatory environment, may eventually further contribute to AMD progression. Future research is warranted to test the therapeutic potential of compounds that stimulate FH/FHL-1 function and to identify the most suitable patients for FH-related therapies.

## Data Availability

Not applicable.
